# Crystal structure of 9-(di­bromo­meth­yl)-1,1-di­fluoro-3,7-dimethyl-1*H*-[1,3,5,2]oxadi­aza­borinino[3,4-*a*][1,8]naphthyridin-11-ium-1-uide

**DOI:** 10.1107/S2056989016016704

**Published:** 2016-10-25

**Authors:** Bang Zhong Wang, Jun Ping Zhou, Yong Zhou, Jian Song Luo, Shao Ming Chi

**Affiliations:** a College of Chemistry and Chemical Engineering, Yunnan Normal University, Kunming, 650500, People’s Republic of China

**Keywords:** crystal structure, 1,8-naphthyridine BF_2_ complex, hydrogen bonding

## Abstract

The mol­ecule of the title 1,8-naphthyridine-BF_2_ derivative, C_12_H_10_BBr_2_F_2_N_3_O, is located on a mirror plane running parallel to the entire ring system and the attached methyl C atoms. Individual mol­ecules are stacked along the *b-*axis direction. The cohesion in the crystal structure is accomplished by C—H⋯F hydrogen bonds and additional off-set π–π inter­actions [centroid-to-centroid distance = 3.6392 (9) Å, slippage 0.472 Å], leading to the formation of a three-dimensional supra­molecular network.

## Abstract 2

The mol­ecule in the title compound, C_12_H_10_BBr_2_F_2_N_3_O, exhibits point group symmetry *m*.

## Chemical context   

1,8-Naphthyridines are one of the most widely studied naphthyridine derivatives (Quan *et al.*, 2012 ▸). They can exhibit diverse coordination modes and have excellent optical properties or biological activities. They are also widely employed in the synthesis of metal complexes, *e.g*. for the identification of small mol­ecules (Liang *et al.*, 2012 ▸; Tanaka *et al.*, 2012 ▸) or metal cations (Liu *et al.*, 2014 ▸), as luminescent materials and in biomedical fields (Eweas *et al.*, 2014 ▸; Di Braccio *et al.*, 2014 ▸). BF_2_ compounds based on 1,8-naphthyridine ligands are used as fluorescent dyes due to their high fluorescence quantum yields (Zheng *et al.*, 2015 ▸) and high photochemical stabilities. Their characteristic absorption and emission spectra (Wu *et al.*, 2013 ▸; Li *et al.*, 2010 ▸) can be applied in many fields, such as cell imaging, as mol­ecular probes, solar cells and so on (Boens *et al.*, 2012 ▸; Loudet & Burgess, 2007 ▸). However, only a few BF_2_ compounds based on the 1,8-naphthyridine moiety have been described in the literature. In view of their importance, the title compound, 9-(di­bromo­meth­yl)-1,1-di­fluoro-3,7–dimethyl-1*H*-[1,3,5,2]oxadi­aza­borinino[3,4-*a*][1,8]naphthyridin-11-ium-1-uide, was synthesized and structurally characterized by single crystal X-ray diffraction.
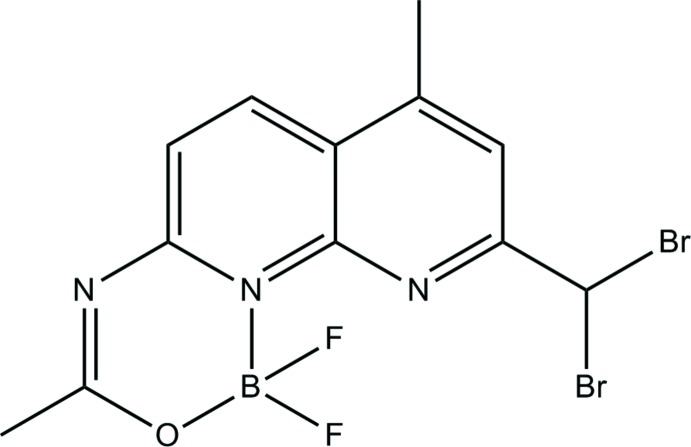


## Structural commentary   

**Figure 1:**
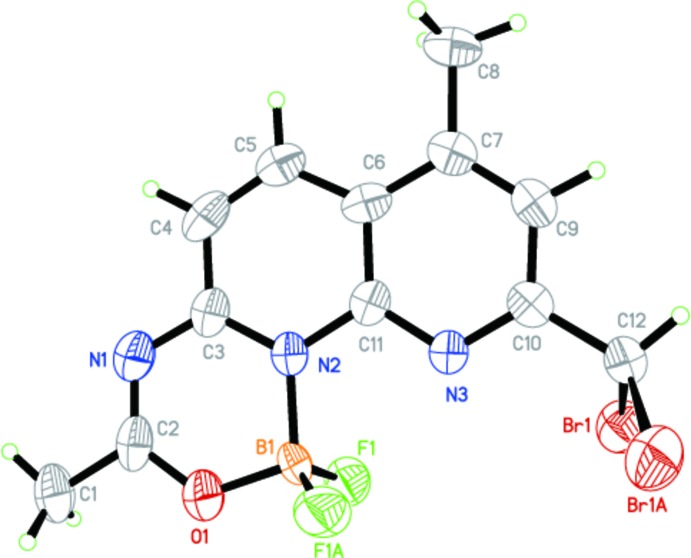
The mol­ecular structure of the title compound, with displacement ellipsoids drawn at the 50% probability level. [Symmetry code: (A) *x*, −*y* + 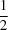, *z*.]

The mol­ecular structure of the title compound is shown in Fig. 1 ▸. The 1,8-naphthyridine ring system is fused with a mixed di­fluoro­roxadi­aza­borinino unit. The entire oxadi­aza­borininona­phthyridine ring system is planar due to its location on a mirror plane running parallel to the ring system. In addition, the C atoms of the two methyl groups (C8 and C1) as well as the C atom (C12) of the di­bromo­methyl group are located on the mirror plane, hence only two pairs of the methyl H atoms, the two Br atoms and the two F atoms are above and below this plane. The F1—B1—F1^i^ and Br1^i^—C12—Br1 angles [symmetry code: (i) *x*, −*y* + 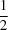, *z*] are 113.6 (7) and 110.3 (3)°, and the distances of the Br and F atoms to the plane are 1.5916 (6) and 1.141 (3) Å, respectively. The individual F—B bond length is 1.364 (5) Å and the Br—C bond length 1.940 (4) Å. Compared with the mol­ecular structure of a related compound (Wu *et al.*, 2012 ▸), the difference between the F1—B1—F1^i^ angles is 2.16°, while the bond lengths and angles in the oxadi­aza­borine ring moiety of the two structures are almost the same.

## Supra­molecular features   

**Figure 2:**
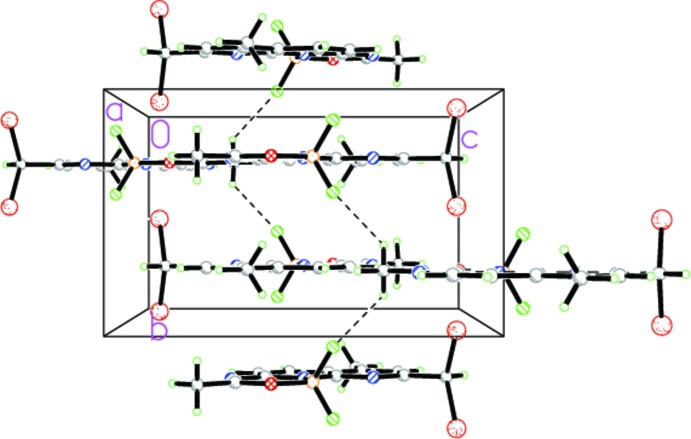
A view along the *a* axis of the crystal packing of the title compound. Hydrogen bonds are shown as dashed lines.

**Figure 3:**
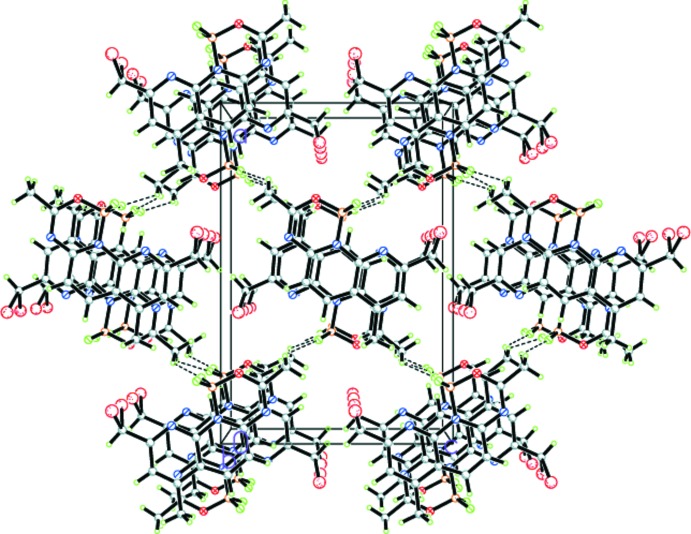
A view along the *b* axis of the crystal packing of the title compound. Hydrogen bonds are shown as dashed lines.

In the crystal structure of the title compound, the mol­ecules are stacked along the *b-*axis direction and linked into a three-dimensional network through C—H⋯F hydrogen bonds involving one of the methyl groups as acceptor H atoms (Fig. 2 ▸, Table 1 ▸). The cohesion in this network is reinforced *via* off-set π–π inter­actions [*Cg*2⋯*Cg*2^i^ = 3.6392 (9) Å, inter­planar distance = 3.6085 (1) Å, slippage = 0.472 Å; *Cg*2 is the centroid of the N2/C3–C6/C11 ring; symmetry code: (i) −*x*, −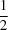 + *y*, 2 − *z*] (Fig. 3 ▸).

## Database survey   

Owing to the shortage of BF_2_ compounds based on 1,8-naphthyridine derivatives, there are only a few examples of similar compounds in the literature. A search of the Cambridge Structural Database (CSD version 5.37; August 19, 2016; Groom *et al.*, 2016 ▸) revealed the structure of another very similar compound, *viz.* [*N*-(5,7-dimethyl-1,8-naphthyridin-2-yl)ethanimidato](di­fluoro)­borate (CSD code MONGED; Du *et al.*, 2014 ▸).

## Synthesis and crystallization   

BF_3_·OEt_2_ (2 ml, 16 mmol) was added dropwise to an ice-cooled solution of 2,6-lutidine (1 ml) and *N*-[7-(di­bromo­meth­yl)-5-methyl-1,8-naphthyridin-2-yl]acetamide (0.37 g, 1 mmol) in anhydrous CH_2_Cl_2_ (80 ml) under a nitro­gen atmosphere. After the mixture had been stirred for 24 h under ambient temperature, the reaction was quenched with 20 ml distilled water. The aqueous layer was extracted with CH_2_Cl_2_ (3 × 50 ml); the organic layer was dried with anhydrous Na_2_SO_4_ and the solvent removed under reduced pressure. The residue was purified by silica gel chromatography using CH_2_Cl_2_ as eluent to give the pure product as a bright white powder (yield 0.19 g, 45%). Yellow crystals of the title compound were obtained from its CH_2_Cl_2_ solution by slow evaporation at room temperature.

## Refinement   

Crystal data, data collection and structure refinement details are summarized in Table 2 ▸. H atoms were placed in calculated positions and included in the final cycles of refinement using a riding-model approximation with C—H = 0.96 Å and with *U*_iso_(H) = 1.2*U*_eq_(C) for aromatic and *U*_iso_(H) = 1.5*U*_eq_(C) for methyl H atoms.

## Supplementary Material

Crystal structure: contains datablock(s) I. DOI: 10.1107/S2056989016016704/wm5325sup1.cif

Structure factors: contains datablock(s) I. DOI: 10.1107/S2056989016016704/wm5325Isup2.hkl

Supporting information file. DOI: 10.1107/S2056989016016704/wm5325Isup3.cml

CCDC reference: 1510400

Additional supporting information: crystallographic information; 3D view; checkCIF report

## Figures and Tables

**Table 1:** Hydrogen-bond geometry (Å, °)

*D*—H⋯*A*	*D*—H	H⋯*A*	*D*⋯*A*	*D*—H⋯*A*
C1—H1*B*⋯F1^i^	0.96	2.41	3.163 (6)	135

Symmetry code: (i) 
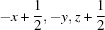
.

**Table 2:** Experimental details

Crystal data	
Chemical formula	C_12_H_10_BBr_2_F_2_N_3_O
*M*_r_	420.86
Crystal system, space group	Orthorhombic, *P* *n* *m* *a*
Temperature (K)	293
*a*, *b*, *c* (Å)	17.161 (3), 7.2169 (14), 11.678 (2)
*V* (Å^3^)	1446.3 (5)
*Z*	4
Radiation type	Mo *K*α
μ (mm^−1^)	5.63
Crystal size (mm)	0.32 × 0.30 × 0.28

Data collection	
Diffractometer	Rigaku R-AXIS RAPID
Absorption correction	Multi-scan (*ABSCOR*; Higashi, 1995 ▸)
*T*_min_, *T*_max_	0.266, 0.302
No. of measured, independent and observed [*I* > 2σ(*I*)] reflections	13517, 1765, 937
*R*_int_	0.139
(sin θ/λ)_max_ (Å^−1^)	0.647

Refinement	
*R*[*F* ^2^ > 2σ(*F* ^2^)], *wR*(*F* ^2^), *S*	0.054, 0.120, 0.95
No. of reflections	1765
No. of parameters	122
H-atom treatment	H-atom parameters constrained
Δρ_max_, Δρ_min_ (e Å^−3^)	0.50, −0.37

Computer programs: *PROCESS-AUTO* (Rigaku, 1998 ▸), *CrystalStructure* (Rigaku/MSC, 2006 ▸), *SHELXS97*, *SHELXL97* and *XP* in *SHELXTL* (Sheldrick, 2008 ▸).

## supplementary crystallographic information

### Crystal data

**Table:**

C_12_H_10_BBr_2_F_2_N_3_O	*F*(000) = 816
*M**_r_* = 420.86	*D*_x_ = 1.933 Mg m^−^^3^
Orthorhombic, *P**n**m**a*	Mo *K*α radiation, λ = 0.71073 Å
Hall symbol: -P 2ac 2n	Cell parameters from 1765 reflections
*a* = 17.161 (3) Å	θ = 3.1–26.0°
*b* = 7.2169 (14) Å	µ = 5.63 mm^−^^1^
*c* = 11.678 (2) Å	*T* = 293 K
*V* = 1446.3 (5) Å^3^	Block, yellow
*Z* = 4	0.32 × 0.30 × 0.28 mm

### Data collection

**Table:**

Rigaku R-AXIS RAPID diffractometer	1765 independent reflections
Radiation source: fine-focus sealed tube	937 reflections with *I* > 2σ(*I*)
Graphite monochromator	*R*_int_ = 0.139
ω scans	θ_max_ = 27.4°, θ_min_ = 3.3°
Absorption correction: multi-scan (*ABSCOR*; Higashi, 1995)	*h* = −22→22
*T*_min_ = 0.266, *T*_max_ = 0.302	*k* = −8→9
13517 measured reflections	*l* = −15→15

### Refinement

**Table:**

Refinement on *F*^2^	Secondary atom site location: difference Fourier map
Least-squares matrix: full	Hydrogen site location: inferred from neighbouring sites
*R*[*F*^2^ > 2σ(*F*^2^)] = 0.054	H-atom parameters constrained
*wR*(*F*^2^) = 0.120	*w* = 1/[σ^2^(*F*_o_^2^) + (0.0519*P*)^2^] where *P* = (*F*_o_^2^ + 2*F*_c_^2^)/3
*S* = 0.95	(Δ/σ)_max_ < 0.001
1765 reflections	Δρ_max_ = 0.50 e Å^−^^3^
122 parameters	Δρ_min_ = −0.37 e Å^−^^3^
0 restraints	Extinction correction: SHELXL, Fc^*^=kFc[1+0.001xFc^2^λ^3^/sin(2θ)]^-1/4^
Primary atom site location: structure-invariant direct methods	Extinction coefficient: 0.0042 (6)

### Special details

**Table:**

Geometry. All esds (except the esd in the dihedral angle between two l.s. planes) are estimated using the full covariance matrix. The cell esds are taken into account individually in the estimation of esds in distances, angles and torsion angles; correlations between esds in cell parameters are only used when they are defined by crystal symmetry. An approximate (isotropic) treatment of cell esds is used for estimating esds involving l.s. planes.
Refinement. Refinement of F^2^ against ALL reflections. The weighted R-factor wR and goodness of fit S are based on F^2^, conventional R-factors R are based on F, with F set to zero for negative F^2^. The threshold expression of F^2^ > 2sigma(F^2^) is used only for calculating R-factors(gt) etc. and is not relevant to the choice of reflections for refinement. R-factors based on F^2^ are statistically about twice as large as those based on F, and R- factors based on ALL data will be even larger.

### Fractional atomic coordinates and isotropic or equivalent isotropic displacement parameters (Å^2^)

**Table:**

	*x*	*y*	*z*	*U*_iso_*/*U*_eq_	
Br1	0.11449 (3)	0.02946 (8)	0.57510 (5)	0.0624 (3)	
B1	0.1752 (5)	0.2500	0.9765 (7)	0.041 (2)	
F1	0.19639 (16)	0.0919 (4)	0.9205 (3)	0.0585 (8)	
N1	0.1041 (4)	0.2500	1.2022 (6)	0.0524 (16)	
N2	0.0835 (3)	0.2500	1.0005 (5)	0.0349 (13)	
N3	0.0640 (3)	0.2500	0.8049 (5)	0.0385 (14)	
O1	0.2143 (3)	0.2500	1.0882 (5)	0.0558 (14)	
C1	0.2326 (5)	0.2500	1.2869 (8)	0.063 (2)	
H1A	0.2025	0.2500	1.3562	0.095*	
H1B	0.2649	0.1414	1.2847	0.095*	
C2	0.1794 (5)	0.2500	1.1872 (7)	0.0450 (19)	
C3	0.0572 (4)	0.2500	1.1095 (7)	0.0408 (18)	
C4	−0.0249 (5)	0.2500	1.1303 (8)	0.055 (2)	
H4A	−0.0433	0.2500	1.2079	0.065*	
C5	−0.0760 (4)	0.2500	1.0421 (8)	0.049 (2)	
H5A	−0.1309	0.2500	1.0579	0.059*	
C6	−0.0513 (4)	0.2500	0.9274 (8)	0.0417 (18)	
C7	−0.1003 (4)	0.2500	0.8304 (7)	0.0435 (19)	
C8	−0.1880 (4)	0.2500	0.8438 (8)	0.060 (2)	
H8A	−0.2125	0.2500	0.7698	0.089*	
H8B	−0.2036	0.1414	0.8854	0.089*	
C9	−0.0662 (4)	0.2500	0.7256 (7)	0.0463 (19)	
H9A	−0.0981	0.2500	0.6580	0.056*	
C10	0.0160 (4)	0.2500	0.7152 (7)	0.0424 (18)	
C11	0.0314 (4)	0.2500	0.9079 (6)	0.0349 (17)	
C12	0.0518 (4)	0.2500	0.5979 (7)	0.050 (2)	
H12A	0.0104	0.2500	0.5425	0.059*	

### Atomic displacement parameters (Å^2^)

**Table:**

	*U*^11^	*U*^22^	*U*^33^	*U*^12^	*U*^13^	*U*^23^
Br1	0.0685 (4)	0.0646 (4)	0.0542 (5)	0.0055 (3)	0.0040 (3)	−0.0122 (3)
B1	0.037 (5)	0.055 (5)	0.029 (5)	0.000	−0.010 (4)	0.000
F1	0.0538 (17)	0.0646 (18)	0.057 (2)	0.0161 (15)	−0.0013 (15)	−0.0145 (17)
N1	0.062 (4)	0.059 (4)	0.037 (4)	0.000	0.004 (4)	0.000
N2	0.042 (3)	0.033 (3)	0.030 (4)	0.000	−0.002 (3)	0.000
N3	0.040 (3)	0.044 (3)	0.032 (4)	0.000	0.000 (3)	0.000
O1	0.055 (3)	0.076 (4)	0.036 (4)	0.000	−0.006 (3)	0.000
C1	0.079 (6)	0.068 (5)	0.042 (6)	0.000	−0.016 (5)	0.000
C2	0.073 (6)	0.031 (4)	0.031 (5)	0.000	−0.003 (4)	0.000
C3	0.056 (5)	0.033 (3)	0.034 (5)	0.000	0.004 (4)	0.000
C4	0.065 (5)	0.053 (4)	0.046 (6)	0.000	0.023 (5)	0.000
C5	0.044 (4)	0.051 (4)	0.053 (6)	0.000	0.011 (4)	0.000
C6	0.037 (4)	0.035 (3)	0.053 (5)	0.000	0.008 (4)	0.000
C7	0.040 (4)	0.036 (4)	0.055 (6)	0.000	−0.002 (4)	0.000
C8	0.036 (4)	0.063 (5)	0.080 (7)	0.000	0.004 (4)	0.000
C9	0.047 (4)	0.047 (4)	0.045 (6)	0.000	−0.009 (4)	0.000
C10	0.043 (4)	0.040 (4)	0.044 (5)	0.000	−0.004 (4)	0.000
C11	0.046 (4)	0.024 (3)	0.035 (5)	0.000	0.004 (3)	0.000
C12	0.046 (4)	0.063 (5)	0.039 (6)	0.000	−0.006 (4)	0.000

### Geometric parameters (Å, º)

**Table:**

Br1—C12	1.940 (4)	C4—C5	1.353 (11)
B1—F1	1.364 (5)	C4—H4A	0.9600
B1—F1^i^	1.364 (5)	C5—C6	1.406 (11)
B1—O1	1.467 (9)	C5—H5A	0.9600
B1—N2	1.599 (10)	C6—C7	1.410 (11)
N1—C2	1.303 (9)	C6—C11	1.437 (9)
N1—C3	1.349 (9)	C7—C9	1.357 (10)
N2—C3	1.351 (9)	C7—C8	1.514 (9)
N2—C11	1.403 (9)	C8—H8A	0.9600
N3—C11	1.327 (8)	C8—H8B	0.9600
N3—C10	1.332 (9)	C9—C10	1.416 (9)
O1—C2	1.302 (9)	C9—H9A	0.9600
C1—C2	1.481 (11)	C10—C12	1.501 (10)
C1—H1A	0.9600	C12—Br1^i^	1.940 (4)
C1—H1B	0.9600	C12—H12A	0.9600
C3—C4	1.431 (10)		
			
F1—B1—F1^i^	113.6 (7)	C6—C5—H5A	118.7
F1—B1—O1	107.7 (4)	C5—C6—C7	125.8 (6)
F1^i^—B1—O1	107.7 (4)	C5—C6—C11	116.7 (7)
F1—B1—N2	110.2 (4)	C7—C6—C11	117.5 (7)
F1^i^—B1—N2	110.2 (4)	C9—C7—C6	117.8 (7)
O1—B1—N2	107.1 (6)	C9—C7—C8	121.5 (7)
C2—N1—C3	118.9 (7)	C6—C7—C8	120.7 (7)
C3—N2—C11	120.9 (6)	C7—C8—H8A	110.0
C3—N2—B1	119.6 (6)	C7—C8—H8B	109.2
C11—N2—B1	119.5 (6)	H8A—C8—H8B	109.5
C11—N3—C10	116.9 (6)	C7—C9—C10	120.5 (7)
C2—O1—B1	125.4 (6)	C7—C9—H9A	119.7
C2—C1—H1A	109.3	C10—C9—H9A	119.8
C2—C1—H1B	109.5	N3—C10—C9	123.2 (7)
H1A—C1—H1B	109.5	N3—C10—C12	117.7 (6)
N1—C2—O1	125.2 (7)	C9—C10—C12	119.1 (7)
N1—C2—C1	120.4 (8)	N3—C11—N2	115.5 (6)
O1—C2—C1	114.4 (7)	N3—C11—C6	124.0 (7)
N2—C3—N1	123.9 (7)	N2—C11—C6	120.5 (7)
N2—C3—C4	119.2 (7)	C10—C12—Br1^i^	110.6 (3)
N1—C3—C4	116.9 (7)	C10—C12—Br1	110.6 (3)
C5—C4—C3	120.7 (8)	Br1^i^—C12—Br1	110.3 (3)
C5—C4—H4A	120.4	C10—C12—H12A	108.2
C3—C4—H4A	119.0	Br1^i^—C12—H12A	108.5
C4—C5—C6	122.0 (7)	Br1—C12—H12A	108.5
C4—C5—H5A	119.3		

Symmetry code: (i) *x*, −*y*+1/2, *z*.

### Hydrogen-bond geometry (Å, º)

**Table:**

*D*—H···*A*	*D*—H	H···*A*	*D*···*A*	*D*—H···*A*
C1—H1*B*···F1^ii^	0.96	2.41	3.163 (6)	135

Symmetry code: (ii) −*x*+1/2, −*y*, *z*+1/2.

## References

[bb1] Boens, N., Leen, V. & Dehaen, W. (2012). *Chem. Soc. Rev.* **41**, 1130–1172. 10.1039/c1cs15132k 21796324

[bb2] Di Braccio, M., Grossi, G., Alfei, S., Ballabeni, V., Tognolini, M., Flammini, L., Giorgio, C., Bertoni, S. & Barocelli, E. (2014). *Eur. J. Med. Chem.* **86**, 394–405. 10.1016/j.ejmech.2014.08.069 25194932

[bb3] Du, M. L., Hu, C. Y., Wang, L. F., Li, C., Han, Y. Y., Gan, X., Chen, Y., Mu, W. H., Huang, M. L. & Fu, W. F. (2014). *Dalton Trans.* **43**, 13924–13931. 10.1039/c4dt01735h 25111133

[bb4] Eweas, A. F., Khalifa, N. M., Ismail, N. S., Al-Omar, M. A. & Soliman, A. M. M. (2014). *Med. Chem. Res.* **23**, 76–86.

[bb5] Groom, C. R., Bruno, I. J., Lightfoot, M. P. & Ward, S. C. (2016). *Acta Cryst.* B**72**, 171–179. 10.1107/S2052520616003954 PMC4822653 27048719

[bb18] Higashi, T. (1995). *ABSCOR*. Rigaku Corporation, Tokyo, Japan.

[bb6] Li, H. J., Fu, W. F., Li, L., Gan, X., Mu, W. H., Chen, W. Q., Duan, X. M. & Song, H. B. (2010). *Org. Lett.* **12**, 2924–2927. 10.1021/ol1003725 20509633

[bb7] Liang, F., Lindsay, S. & Zhang, P. (2012). *Org. Biomol. Chem.* **10**, 8654–8659. 10.1039/c2ob26529j PMC3748945 23038027

[bb8] Liu, X. J., Chen, M. X., Liu, Z. P., Yu, M. M., Wei, L. H. & Li, Z. X. (2014). *Tetrahedron*, **70**, 658–663.

[bb9] Loudet, A. & Burgess, K. (2007). *Chem. Rev.* **107**, 4891–4932. 10.1021/cr078381n 17924696

[bb10] Quan, L., Chen, Y., Lv, X. J. & Fu, W. F. (2012). *Chem. Eur. J.* **18**, 14599–14604. 10.1002/chem.201201592 23032933

[bb11] Rigaku (1998). *PROCESS-AUTO*. Rigaku Corporation, Tokyo, Japan.

[bb12] Rigaku/MSC (2006). *CrystalStructure*. Rigaku/MSC, The Woodlands, Texas, USA.

[bb13] Sheldrick, G. M. (2008). *Acta Cryst.* A**64**, 112–122. 10.1107/S0108767307043930 18156677

[bb14] Tanaka, K., Murakami, M., Jeon, J.-H. & Chujo, Y. (2012). *Org. Biomol. Chem.* **10**, 90–95. 10.1039/c1ob06630g 22041904

[bb15] Wu, Y. Y., Chen, Y., Gou, G. Z., Mu, W. H., Lv, X. J., Du, M. L. & Fu, W. F. (2012). *Org. Lett.* **14**, 5226–5229. 10.1021/ol302347m 23050580

[bb16] Wu, Y. Y., Chen, Y., Mu, W. H., Lv, X. J. & Fu, W. F. (2013). *J. Photochem. Photobiol. Chem.* **272**, 73–79.

[bb17] Zheng, J., Huang, F., Li, Y. J., Xu, T. W., Xu, H., Jia, J. H., Ye, Q. & Gao, J. R. (2015). *Dyes Pigments*, **113**, 502–509.

